# Mechanical Recycling of Post-Consumer Polypropylene Fishing Nets: Effect of Blending with Virgin Polymer

**DOI:** 10.3390/polym18182232

**Published:** 2026-09-13

**Authors:** Erika Indovino, Francesco Paolo La Mantia, Luigi Botta

**Affiliations:** 1 Department of Engineering, University of Palermo, Viale delle Scienze, 90128 Palermo, Italy; erika.indovino@unipa.it; 2 National Interuniversity Consortium of Materials Science and Technology (INSTM), Via Giusti 9, 50121 Florence, Italy

**Keywords:** post-consumer polypropylene, fishing nets, mechanical recycling, circular economy, recycled/virgin blends

## Abstract

The extensive use of plastic materials across the fisheries sector, including fishing, aquaculture and fish product manufacturing and processing, represents a significant source of environmental pollution. In particular, end-of-life fishing nets, typically made of oil-based polymers, contribute to the accumulation of persistent plastic waste in marine ecosystems. Mechanical recycling has recently emerged as a promising strategy to recover such materials and reintroduce them into production cycles, supporting circular economy practices. In this work, post-consumer polypropylene (PP) fishing nets were collected and mechanically recycled. The recovered polymer was blended with virgin PP at 10 wt% and 30 wt% and processed via extrusion. The resulting materials were characterized in terms of rheological, structural, and mechanical properties to assess their processability and performance. The results demonstrate that recycled PP derived from fishing nets can be effectively reprocessed and used as a secondary raw material, enabling the development of more sustainable polymer systems while reducing dependence on virgin plastics and mitigating environmental impact.

## 1. Introduction

The accumulation of plastic waste derived from fishing activities represents a critical environmental and technological issue within the framework of the circular economy [1]. It is estimated that approximately 640,000 tonnes of fishing nets are abandoned, lost, or discarded annually in marine environments, while in Europe these materials constitute nearly 27% of total marine litter [2,3]. Environmental monitoring campaigns have consistently demonstrated that a substantial proportion of seabed debris originates from fisheries-related activities, with fishing nets, ropes and lines representing predominant components in both coastal and offshore ecosystems [4]. In addition to their severe ecological impact on marine biodiversity, abandoned fishing nets contribute to the long-term accumulation of persistent polymeric materials, resulting in both environmental degradation and the irreversible loss of valuable resources.

Fishing nets are predominantly manufactured from high-performance synthetic polymers, including nylon 6 (PA6), nylon 66 (PA66), high-density polyethylene (HDPE), polypropylene (PP), and poly(ethersulfone) (PES) [5]. These materials are specifically selected owing to their outstanding mechanical strength, abrasion resistance, thermal stability and hydrophobic properties, which are necessary to ensure reliable performance in severe marine conditions over extended periods. However, their resistance to degradation leads to long-term persistence in marine environments, contributing to ecological damage and the loss of valuable polymeric resources.

In response to these concerns, considerable research efforts have been directed toward the development of more sustainable alternatives and recovery strategies. One major research direction involves the introduction of biodegradable and bio-based polymers, such as poly(lactic acid) (PLA), poly(butylene succinate) (PBS), poly(butylene adipate terephthalate) (PBAT), and poly(hydroxyalkanoates) (PHA) [6,7,8]. These materials are frequently enhanced through advanced engineering approaches, including nanocomposite formulations and bioinspired fiber architectures, with the aim of improving their mechanical and functional performance. Consequently, contemporary fishing nets are increasingly evolving into highly engineered systems designed to reconcile mechanical durability with environmental sustainability [9]. Nevertheless, despite significant technological advancements, the implementation of biodegradable polymers in the fisheries sector remains limited due to inadequate long-term durability and insufficient reliability under real operational conditions. Table 1 summarizes the main characteristics of synthetic and biodegradable polymers commonly used in fishing nets, as previously mentioned.

Concurrently, increasing attention has been devoted to the recycling and valorization of post-consumer fishing nets. Among the available approaches, mechanical recycling currently represents the most established and industrially adopted strategy. This process generally involves grinding, melting and pelletizing operations to convert recovered polymeric waste into reusable secondary raw materials. However, polymers retrieved from marine environments are frequently affected by extensive degradation phenomena, including ultraviolet-induced oxidation, hydrolytic degradation, mechanical wear and contamination by inorganic particles and biological residues. Such factors can substantially impair the rheological, thermal and mechanical properties of recycled materials, thereby limiting their direct reutilization in high-performance applications.

Chemical recycling technologies have also been extensively investigated. In particular, depolymerization processes enable the recovery of monomeric species from polymeric waste streams, facilitating the regeneration of materials with properties comparable to virgin polymers. For example, thermocatalytic depolymerization of PA6 into caprolactam has been reported to achieve conversion yields exceeding 80% [10]. Furthermore, several industrial-scale initiatives have demonstrated the technical and economic feasibility of polymer regeneration from end-of-life fishing nets. Regenerated nylon derived from discarded fishing gear is currently employed in textile, construction, and automotive sectors. Notably, companies such as Aquafil produce regenerated Econyl^®^ fibers through advanced polymer recovery technologies [11], while initiatives such as Circulen-Recover^®^ aim to integrate recycled polymers into industrial applications compliant with End-of-Life Vehicle regulations [12].

An additional strategy that has attracted considerable interest involves blending recycled polymers with virgin polymer matrices (monopolymer blends) in order to compensate for the deterioration of material properties associated with environmental aging and degradation [13,14,15]. Within this scientific and technological context, the present work proposes a sustainable materials engineering strategy based on blending recycled polymers derived from abandoned fishing nets with a controlled fraction of virgin polymer. The proposed approach aims to preserve adequate mechanical and processing performance while simultaneously reducing the consumption of primary fossil-based raw materials. Furthermore, the valorization of post-consumer fishing nets contributes directly to the mitigation of marine plastic pollution and promotes the reintegration of waste-derived polymers into the production cycle. Overall, the proposed methodology supports the transition toward a more sustainable and circular management of plastic resources through increased recycling efficiency, enhanced material recovery and reduced dependence on virgin polymer feedstocks.

## 2. Materials and Methods

**Figure 1:**
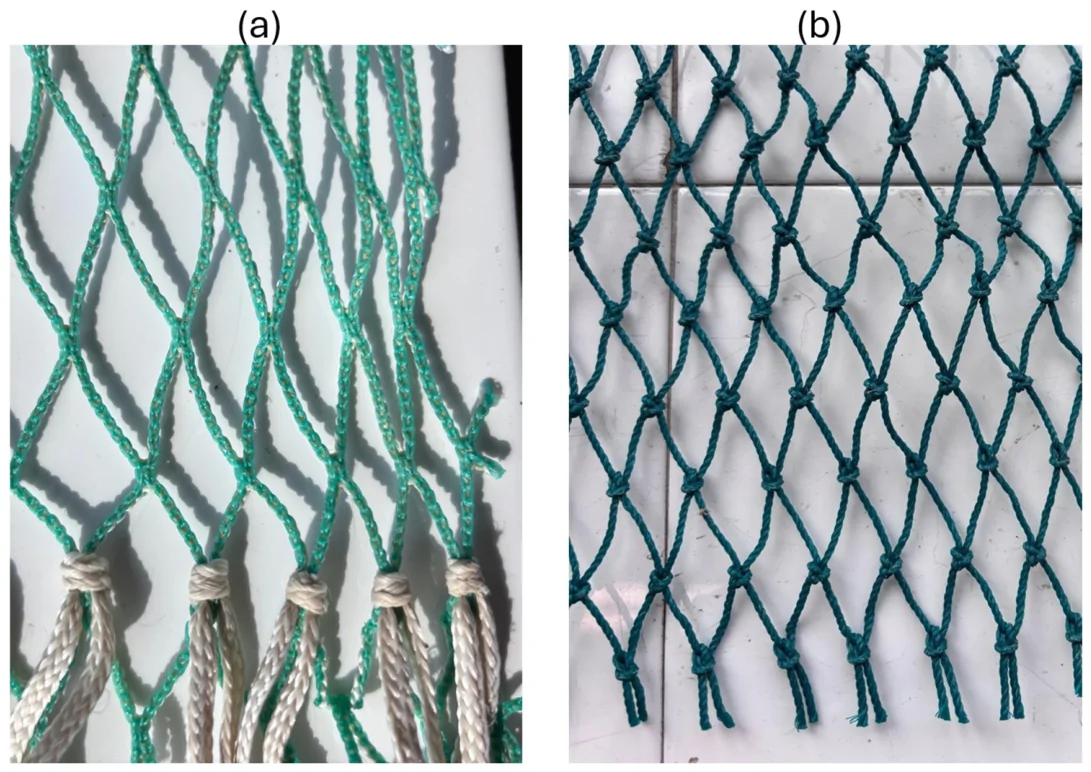
Post-consumer fishing nets as received: (**a**) NET_1; (**b**) NET_2.

The materials used in this work were a commercial polypropylene (PP) sample known as Moplen^®^ RP340H, LyondellBasell (Ferrara, Italy), having a melt flow rate (MFR) equal to 1.8 g/10 min (at 230 °C and 2.16 kg), and post-consumer fishing nets (PCNs) made up of polypropylene (PP) recovered from a local fishing fleet of Sciacca (Sicily, Italy). Figure 1 shows the post-consumer fishing nets as received, prior to any processing.

The post-consumer fishing nets were first melt compounded using a Brabender^®^ type mixer (Brabender model PLE330, Duisburg, Germany) at 60 rpm and 190 °C for 5 min in order to obtain pellets and ensure proper homogenization of the material from two different nets. Subsequently, the blends of post-consumer nets with virgin PP (10%wt and 30%wt of PCN) were processed in a laboratory single-screw extruder (Thermo Scientific HAAKE PolyLab QC, Karlsruhe, Germany, D = 19 mm, L/D = 25), operating at a die head temperature of 190 °C and a screw speed of 60 rpm.

All samples were compression molded into sheets at 190 °C for about 3 min under a pressure of approximately 100 bar using a Carver press (Wabash, IN, USA).

**Figure 2:**
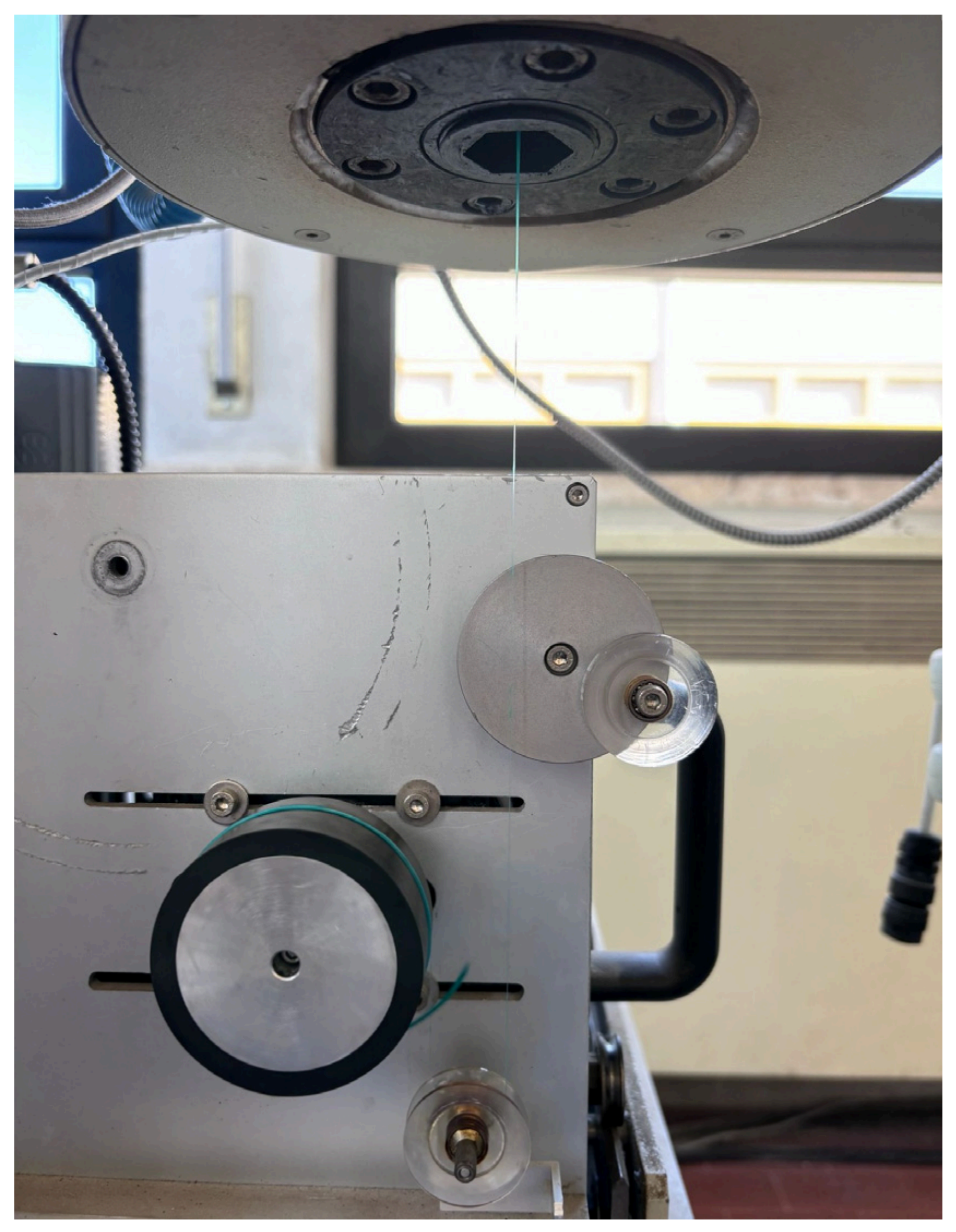
Spinning system of the capillary rheometer with the obtained filament sample.

Fibers were produced with a capillary viscometer (Rheologic 1000, CEAST, Pianezza, Italy) at an extrusion temperature of 190 °C and piston speed of 2 mm/min using a die with a 1 mm diameter and a length-to-diameter ratio of 40 (L/D = 40). A pulley-based drawing system comprising a series of sequentially arranged pulleys was employed to collect and convey the hot filament, as shown in Figure 2.

Subsequently, filaments with diameters ranging from approximately 65 to 200 µm were obtained from the capillary melt by varying the collection pulley speed, resulting in different draw ratios (DRs).

The draw ratio was calculated as

DR = *D*_0_^2^/*D*_f_^2^ (1)

where *D*_0_ is the capillary diameter, and *D*_*f*_ is the final fiber diameter.

### 2.1. Characterization of the Post-Consumer Nets

#### 2.1.1. Spectra Analysis

All collected post-consumer fishing nets were characterized by FT-IR/ATR analysis using a Perkin-Elmer Spectrum One spectrometer (PerkinElmer, Waltham, MA, USA), with data processed using Spectrum One software (version 10). Specifically, the spectra, averaged over 16 scans, were recorded in the range of 450–4000 cm^−1^.

#### 2.1.2. Thermal Studies

Differential scanning calorimetry (DSC) was carried out using a DSC 131 analyzer (Setaram, Hillsborough Township, NJ, USA). The tests were performed under a nitrogen atmosphere, employing approximately 10–12 mg of sample and applying a heating rate of 10 °C/min up to 250 °C. These preliminary measurements were mainly aimed at confirming the specific polymeric composition of the fishing nets under investigation. The degree of crystallinity (χc) was determined using Equation (2):

(2) $$\chi_{c}=\left(\frac{\Delta H_{m}}{\Delta H_{m}^{\circ }}\right)\times 100$$

where ΔHm represents the melting enthalpy experimentally obtained for each sample, while ΔHm° corresponds to the melting enthalpy of a fully crystalline polymer. For polypropylene (PP), the value of ΔHm° is 207 J/g [16].

#### 2.1.3. Rheological Characterizations

Rheological analyses were conducted using a rotational rheometer (Ares G2, TA Instruments, New Castle, DE, USA) equipped with 25 mm parallel plates in order to characterize the material response at low frequencies. The experiments were performed at 190 °C under a constant strain of 5%, while the angular frequency was varied from 0.1 to 628 rad/s. Five data points were collected for each frequency decade to ensure accurate representation of the viscoelastic behavior.

The non-isothermal extensional rheology of the material was assessed using the capillary viscometer previously described. Measurements were performed at a constant temperature of 190 °C using different piston speeds (1, 2, 5, and 10 mm/min), while the acceleration of the collection pulley was maintained constant at 12 mm/s^2^. Thanks to that apparatus, the force measured at the moment of rupture was defined as the melt strength (MS). The breaking stretching ratio (BSR) was calculated as the ratio between the drawing speed at break and the extrusion velocity at the die exit [17].

#### 2.1.4. Mechanical Characterizations

Mechanical properties were determined through tensile tests carried out with an Instron 3365 Universal Testing Machine (Instron, High Wycombe, UK), following the procedures specified in ASTM D638. The crosshead speed was set to 1 mm/min at the beginning of the test and subsequently increased to 100 mm/min until the specimens fractured. The elastic modulus (E), tensile strength (TS), and elongation at break (EB) were calculated, and the values reported correspond to the average obtained from five samples. Both isotropic sheets and oriented fibers were subjected to mechanical characterization.

All the samples investigated in this work are summarized with their code in Table 2.

## 3. Results and Discussion

### 3.1. Characterization of the Post-Consumer Nets

**Figure 3:**
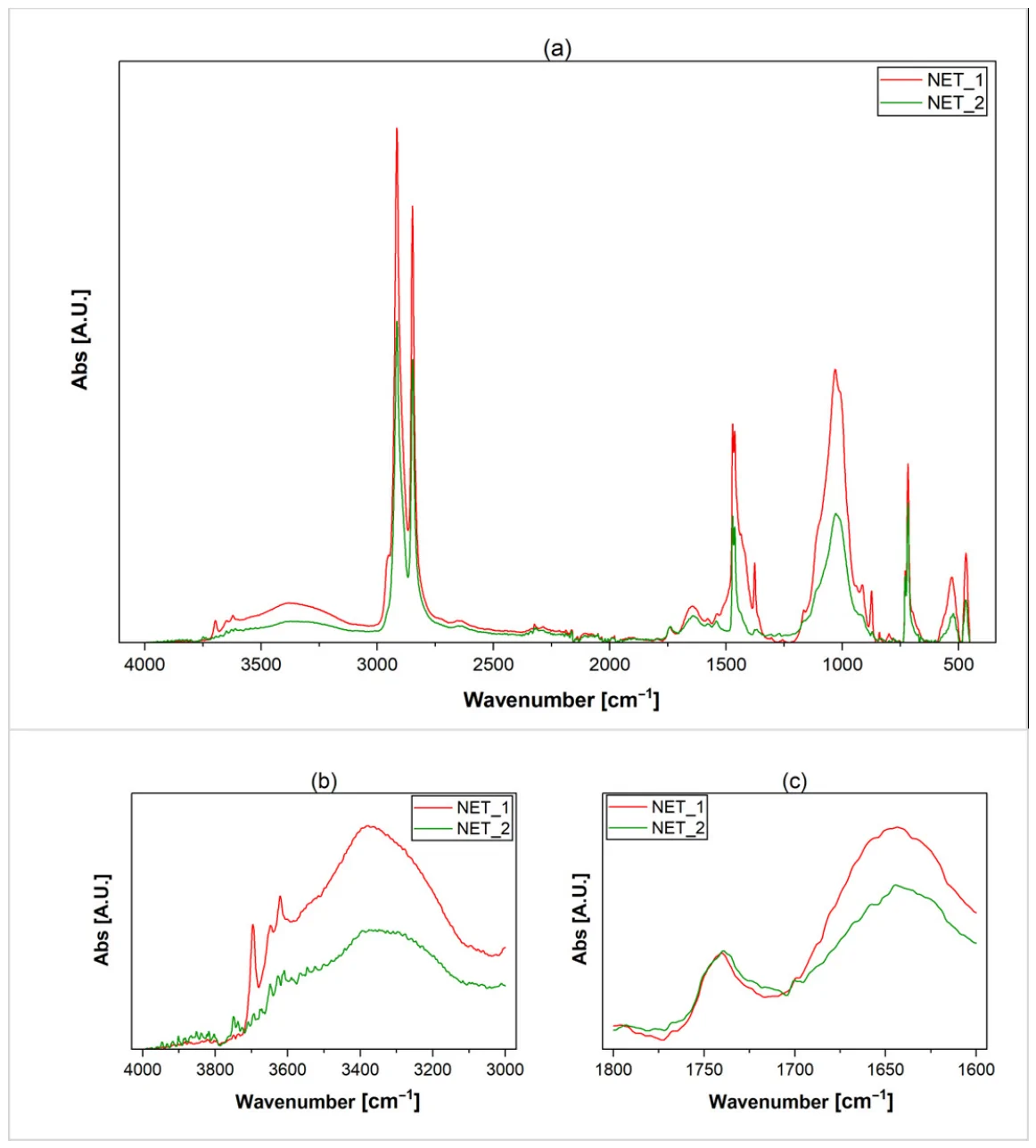
FTIR/ATR spectra of the two samples of nets: (**a**) in the wavenumber range 450–4000 cm^−1^; (**b**) 3000–4000 cm^−1^ and (**c**) 1600–1800 cm^−1^.

In Figure 3, the spectra of the two samples of nets are reported in the full range of 450–4000 cm^−1^ in Figure 3a; in the range of 3000–4000 cm^−1^ in Figure 3b, typical of the oxygenated products resulting from the possible photo-oxidation of these items; and 1600–1800 cm^−1^ in Figure 3c.

It has been demonstrated, indeed, that photo-oxidation can also occur in seawater, although to a lesser extent [18]. A first peak is clearly noted at around 1750 cm^−1^, which indicates the presence of carbonyl compounds [19], and a second peak at about 3400 cm^−1^, which indicates the presence of hydroxyl compounds [20]. These two peaks clearly indicate that the two nets have undergone degradation processes that lead to the presence of oxygenated groups.

The peaks observed at around 2900 cm^−1^ indicate the presence of a certain amount of polyethylene (PE) within the analyzed sample, in particular for NET_2. These signals are typically associated with the C–H stretching vibrations of aliphatic groups, which are characteristic of polyethylene chains. Their detection suggests that PE may be present either as a contaminant or as part of a blended polymer system, likely resulting from mixed plastic waste [21].

In Figure 3a, a significant peak is observed between 900 and 1000 cm^−1^ for both nets. This can be associated with the presence of carbonates due to possible encrustations, as already demonstrated by previous studies [22], since the samples consist of used and discarded fishing nets. Table 3 below reports the mean values of the mechanical properties of the fibers collected from the corresponding NET_1 and NET_2 post-consumer fishing nets.

Tensile properties of filaments extracted from the as-received post-consumer fishing nets revealed comparable mechanical responses. In particular, NET_1 exhibited a slightly higher elastic modulus and tensile strength, accompanied by lower elongation at break. These differences may be related to the slightly higher degree of crystallinity subsequently observed for NET_1, which may increase stiffness by restricting molecular chain mobility and, consequently, reducing fiber deformability.

**Figure 4:**
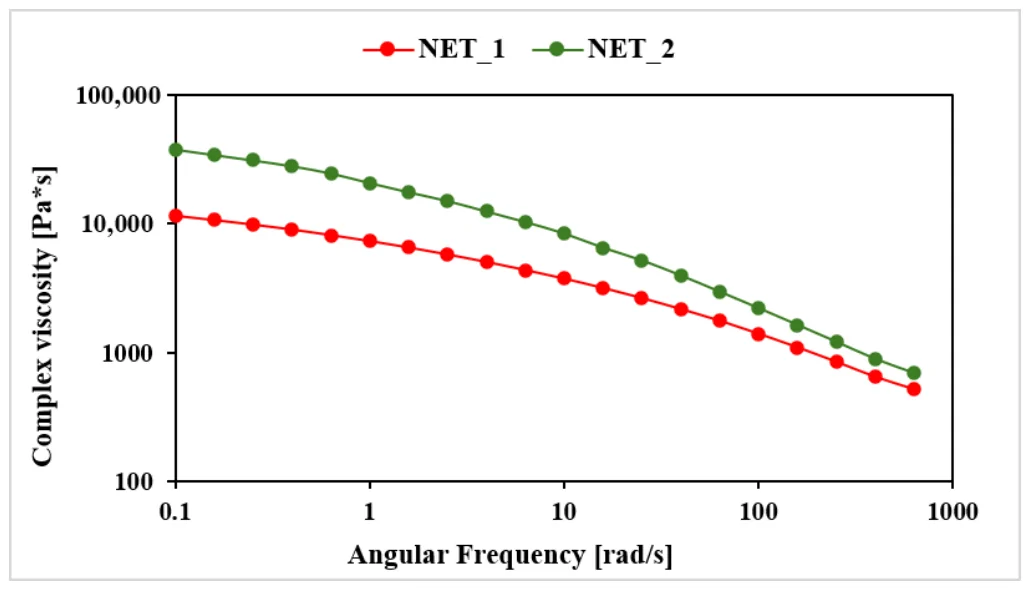
Flow curves of the two samples.

Figure 4 shows the flow curves of the two samples. The flow curve of NET_2 lies above that of NET_1 at low angular frequencies, with the two curves converging in the high-frequency range typical of processing operations; overall, NET_1 shows lower complex viscosity than NET_2.

Both nets display non-Newtonian behavior, which becomes progressively more pronounced with increasing angular frequency. This difference may be attributed either to the different PP grades originally used in the production of the two nets or to differences in their aging history, which can affect the molecular characteristics of the polymer.

**Figure 5:**
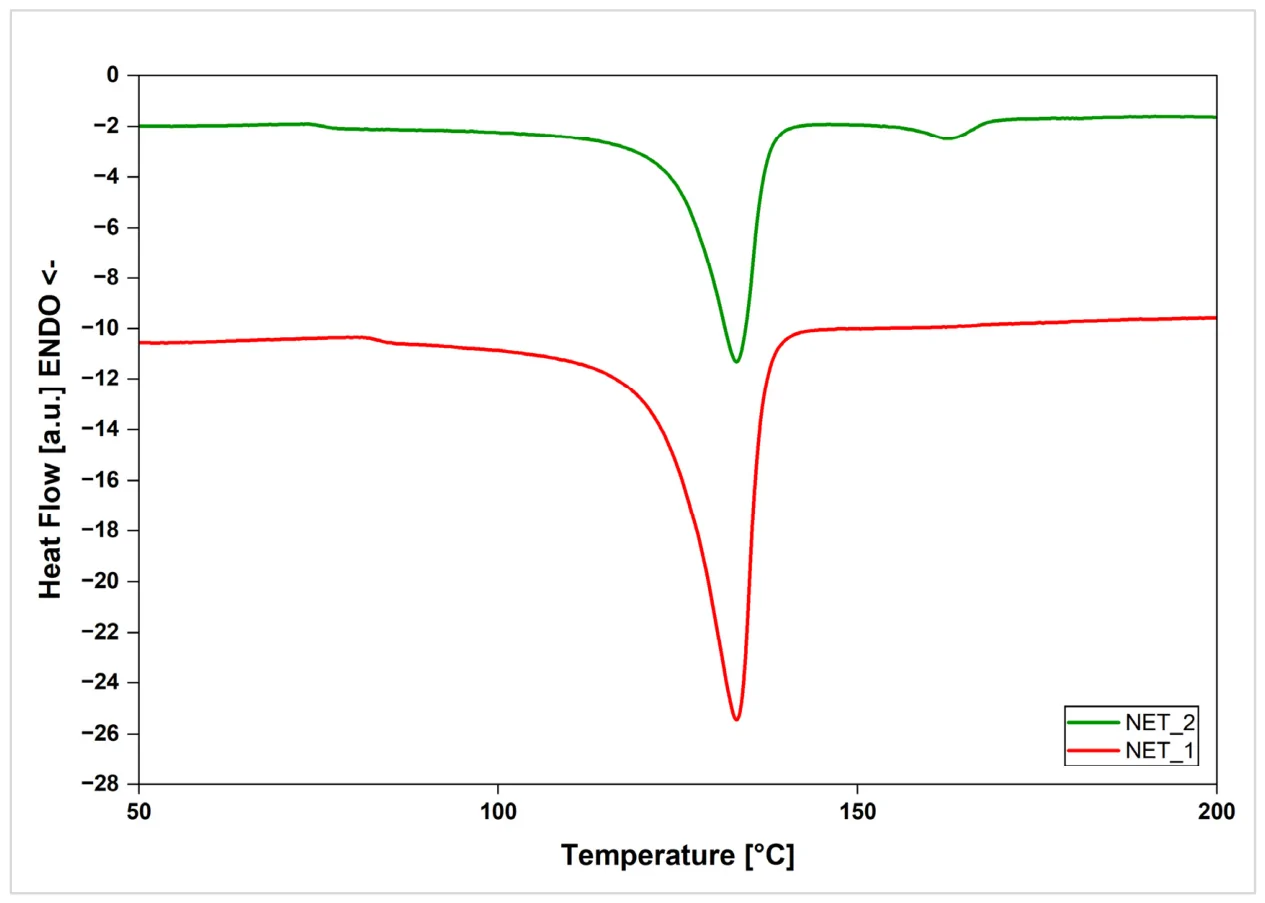
DSC thermograms of the two nets (2nd heating).

The DSC thermograms of the two samples are reported in Figure 5, and the resulting values are reported in Table 4.

It is evident that the two samples are similar but not the same. In particular, the similar melting temperatures of approximately 133 °C observed for both samples suggest that they are PP copolymers with different PE contents, as further supported by their markedly different degrees of crystallinity.

**Figure 6:**
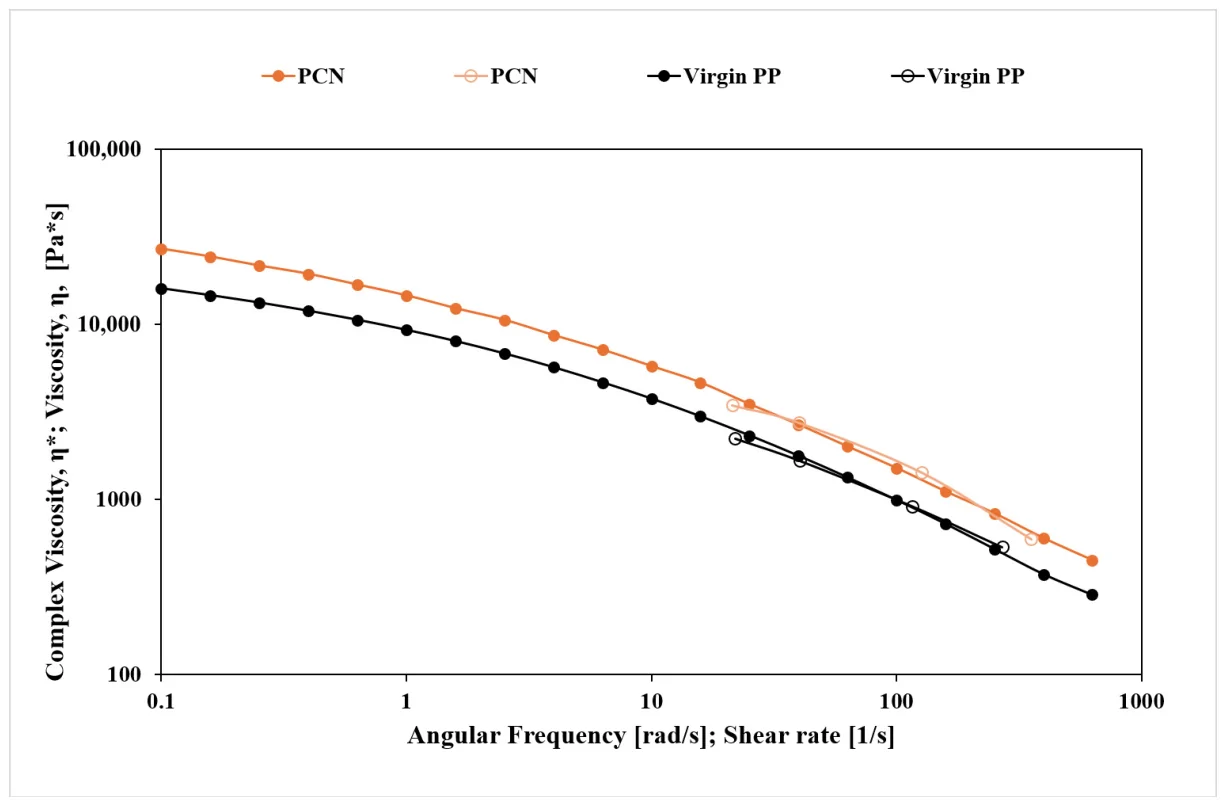
Flow curves under shear flow in the rotational rheometer (closed symbols) and in the capillary viscometer (open symbols) of virgin and post-consumer fishing nets (PCN).

Due to the fact that all the post-consumer nets are collected together, both samples have been processed together (PCN) in equal content (50:50) when mixed with the virgin polymer. And, due to the fact that the exact composition of the virgin polymer is unknown, we decided to use a sample of polypropylene with rheological properties similar to those of the PCN sample. This is in order to have similar processability in the production of the fibers. The flow curves of the post-consumer nets and of the virgin PP are reported in Figure 6. The experimental data obtained in the rotational rheometer and in the capillary viscometer superimpose as expected for these polymers on the basis of the Cox–Merz law [23].

The higher values in complex viscosity of the PCN sample can also be associated with the presence of contaminants, oxidized species, and branched structures generated during aging, during their service life and reprocessing cycles.

Because the fibers are made in a spinning operation where the non-isothermal uniaxial elongation flow is involved, the values of Melt Strength (MS) and Breaking Stretching Ratio (BSR) were analyzed in detail; however, it was not possible to obtain measurable MS and BSR values for the fibers directly produced from the PCN sample because it was not technologically spinnable. This behavior can reasonably be attributed to the presence of contaminants and solid encrustations within the material, which likely acted as structural defects during the spinning and drawing stages. Such heterogeneities may have generated localized stress concentration points along the fiber, causing premature failure and consequently preventing stable elongation and proper stretching of the filaments. For this reason, the recycled fishing nets were blended with virgin polymer PP in order to compensate for this limitation.

### 3.2. Characterization of the Monopolymer Blends

**Figure 7:**
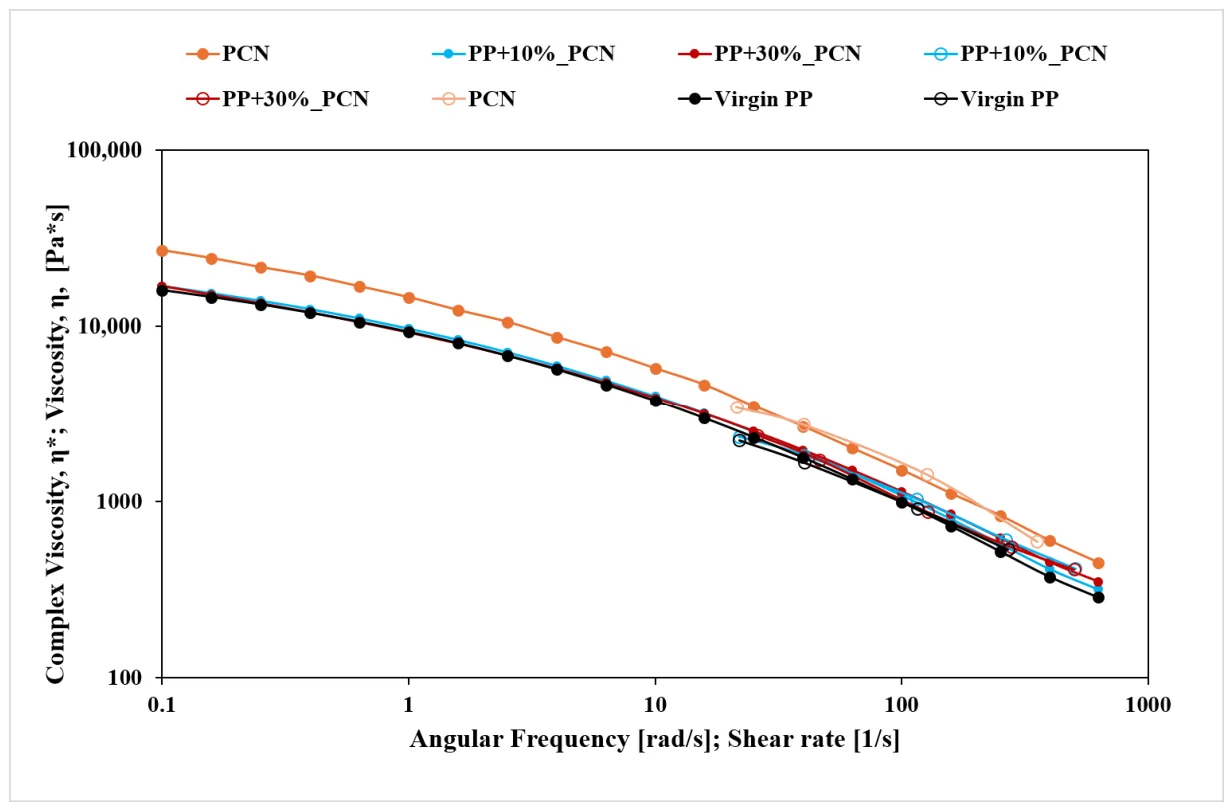
Flow curves under shear flow in the rotational rheometer (closed symbols) and in the capillary viscometer (open symbols) of virgin and post-consumer samples with the monopolymer blends.

The flow curves of the two monopolymer blends are reported in Figure 7 with the flow curves of the two components.

**Figure 8:**
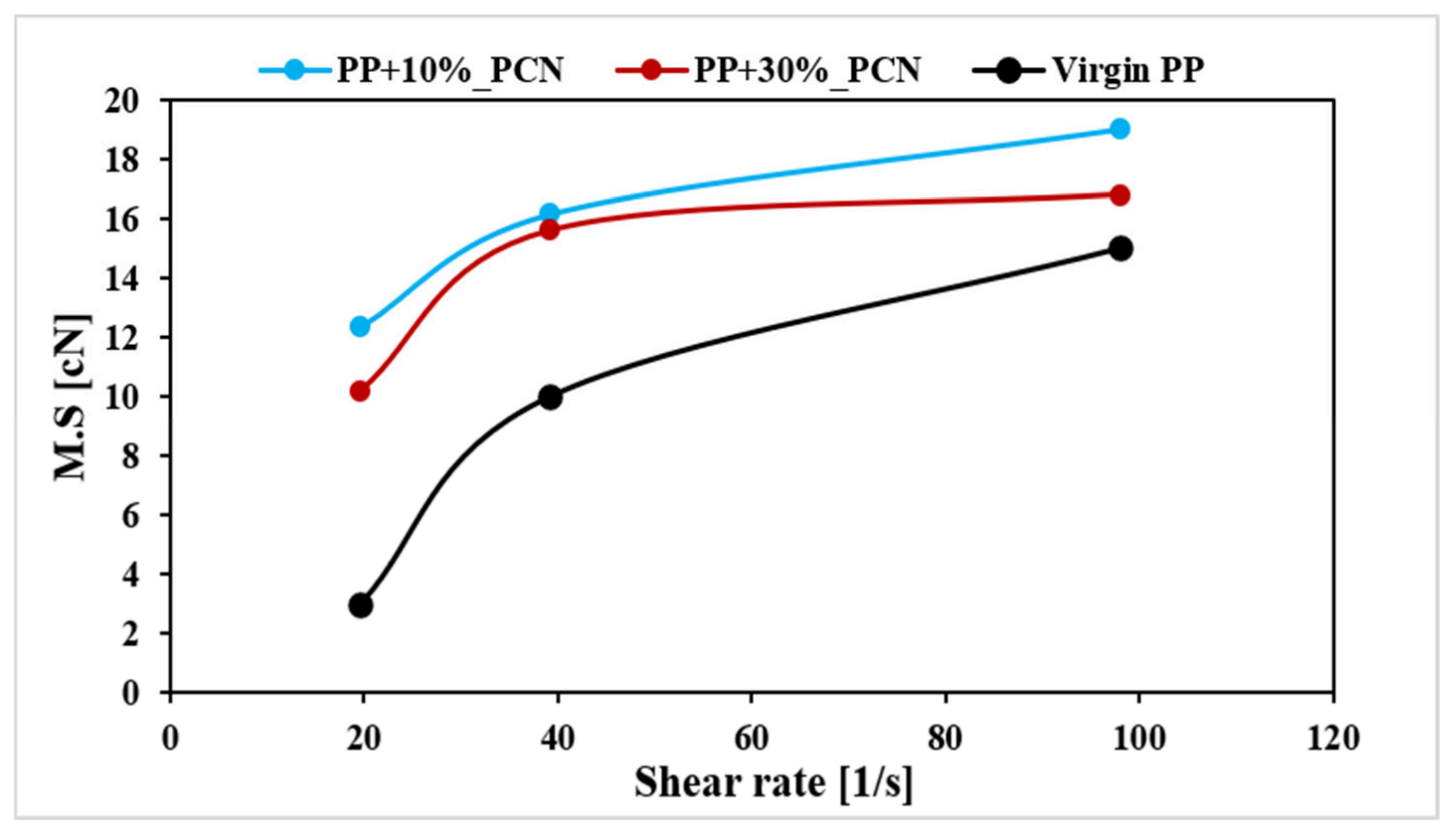
MS values as a function of the apparent shear rate.

**Figure 9:**
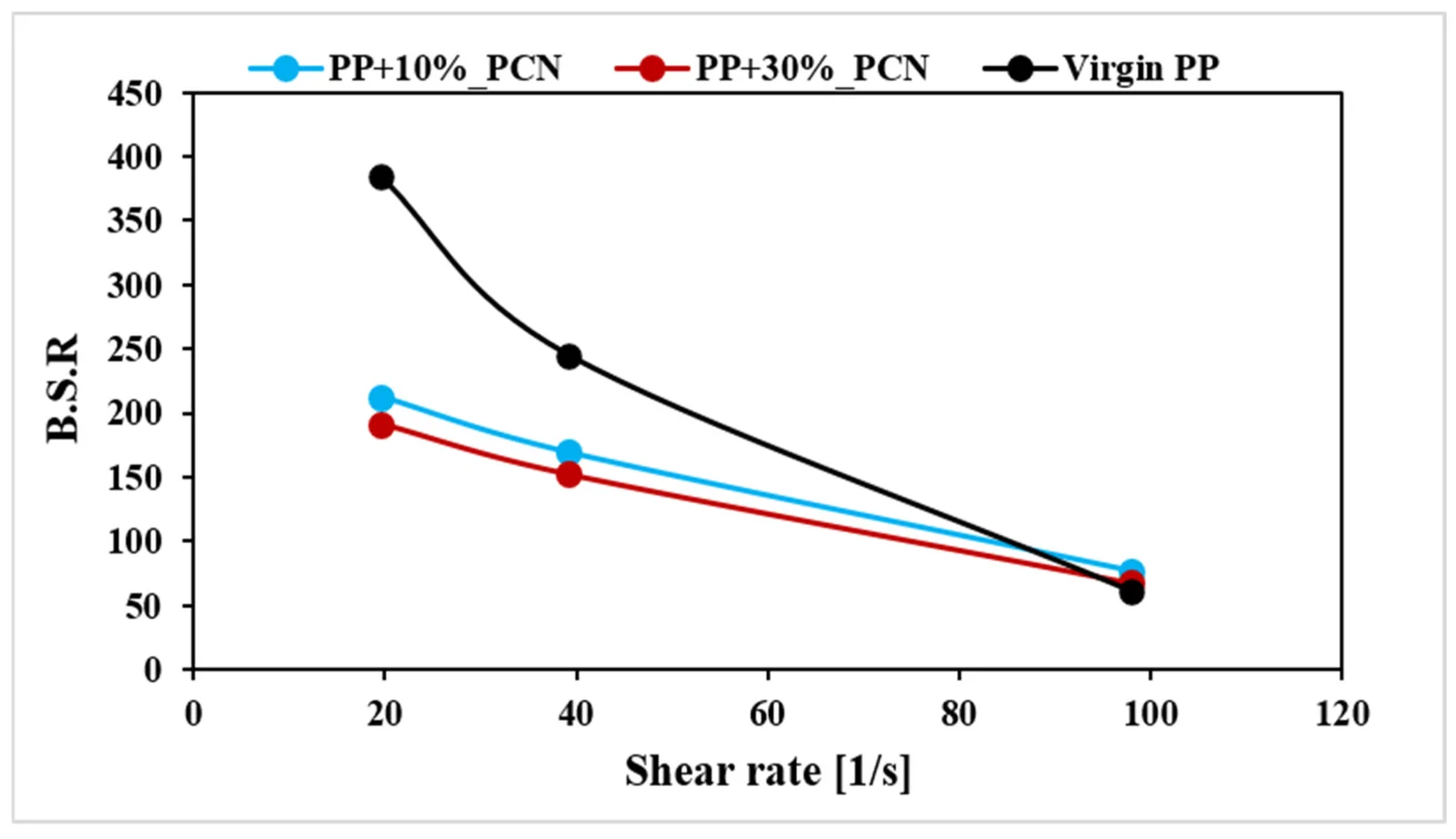
BSR values as a function of the apparent shear rate.

The flow curves of the blends are in between those of the pure components, and all the flow curves tend to gather together at high shear rates. Similar behaviors show both the MS values and the BSR values reported in Figure 8 and Figure 9. In particular, as the content of recycled fishing nets increased, the BSR decreased, while the MS was higher than that of virgin PP because the sample experimentally broke earlier. As previously discussed, this behavior can be attributed to the presence of organic contaminant material. All these rheological experimental results clearly show that the processability both in shear flow and in non-isothermal elongational flow is not worsened in the presence of these investigated contents of post-consumer nets when blended with virgin PP.

**Figure 10:**
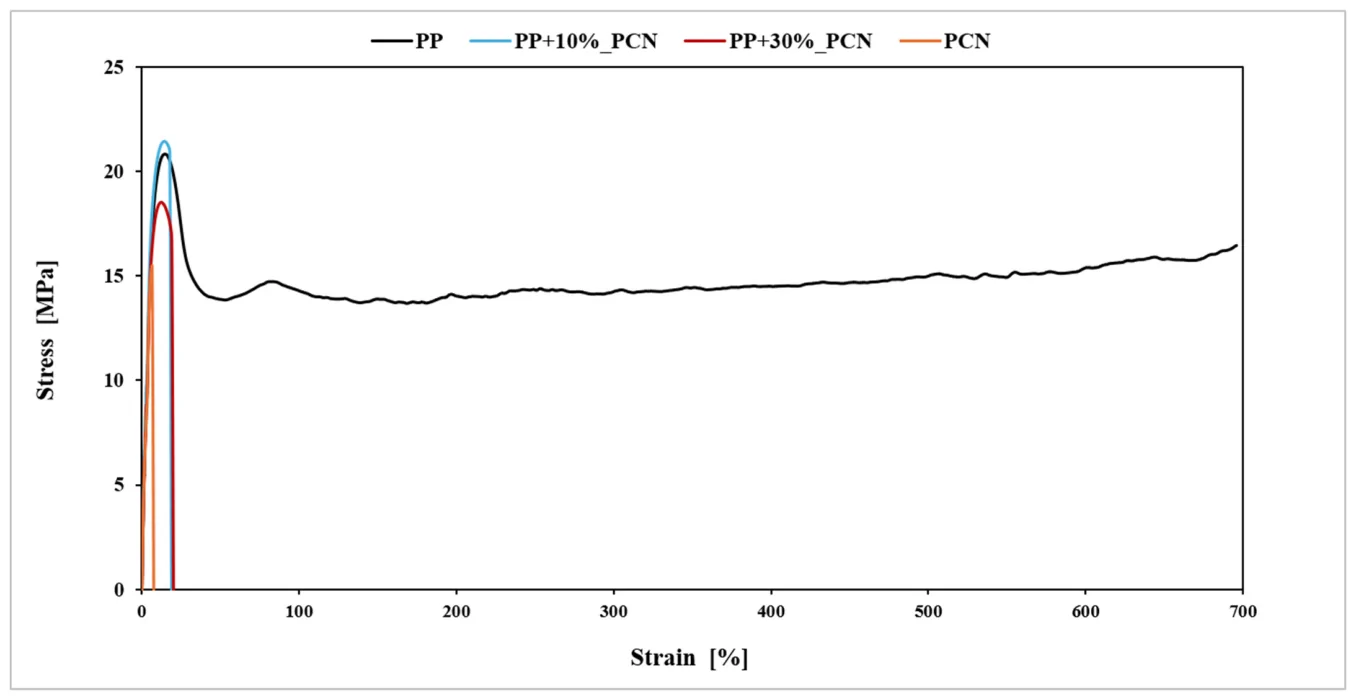
Stress–strain curves of representative specimens of the isotropic sheets.

In Figure 10, the stress–strain curves of representative specimens of the monopolymer blends and of the two components are reported. The corresponding values of the elastic modulus (E), tensile strength (TS), and elongation at break (EB) are reported in Table 5. All measurements were performed on isotropic sheets.

The more impressive difference is certainly in the elongation at break. The post-consumer sample shows brittle behavior, while a similar PP sample is ductile with a very high value of elongation at break. A significant degradation level and, perhaps, the presence of heterogeneities are certainly responsible for this behavior, as reported in other previous papers [24,25,26,27]. Less expected is the fragile behavior of the two blends, considering, in particular, the low content of the brittle component. La Mantia and Valenza [28] have shown that degradation occurring during processing and recycling operations, which modifies the molecular structure of the PP sample, affects the crystalline morphology of PP, leading to different crystalline phases between the virgin and degraded sample. This particular morphology in the solid state gives rise to a sort of “composite” material that is very brittle. This interpretation can explain the low values of the elongation at break and the brittleness of these blends.

**Figure 11:**
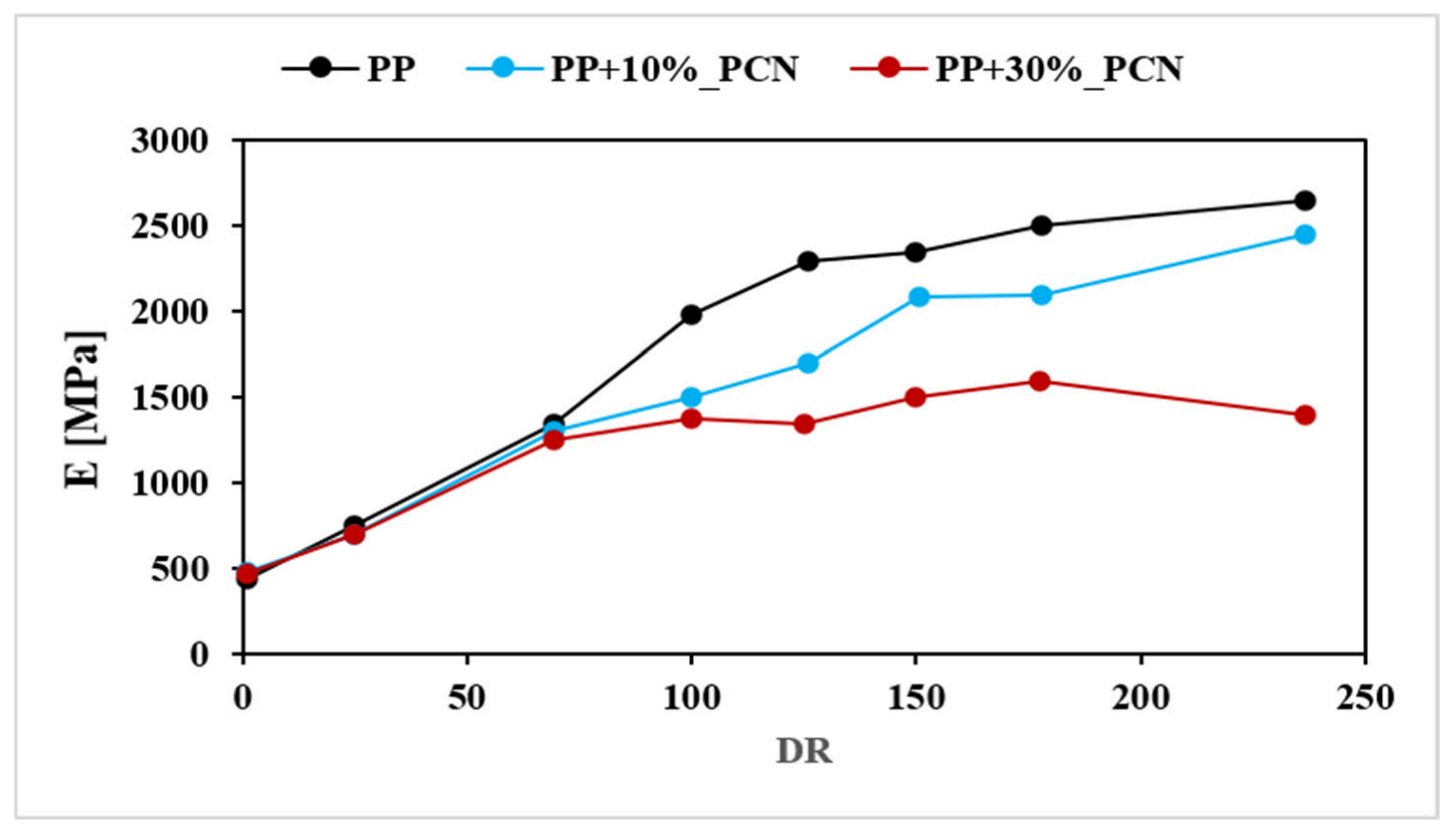
Elastic modulus as a function of the draw ratio.

**Figure 12:**
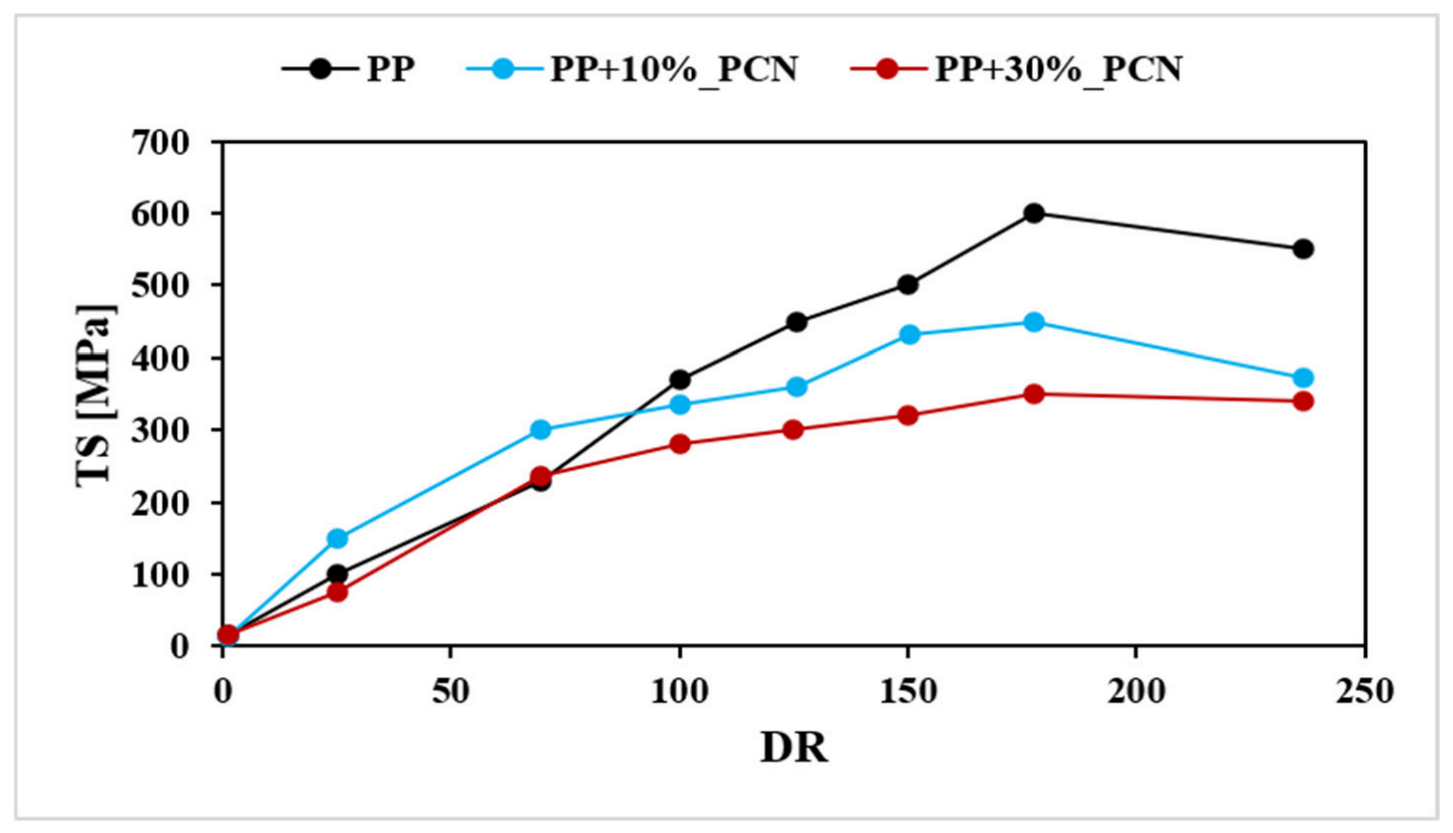
Tensile strength as a function of the draw ratio.

**Figure 13:**
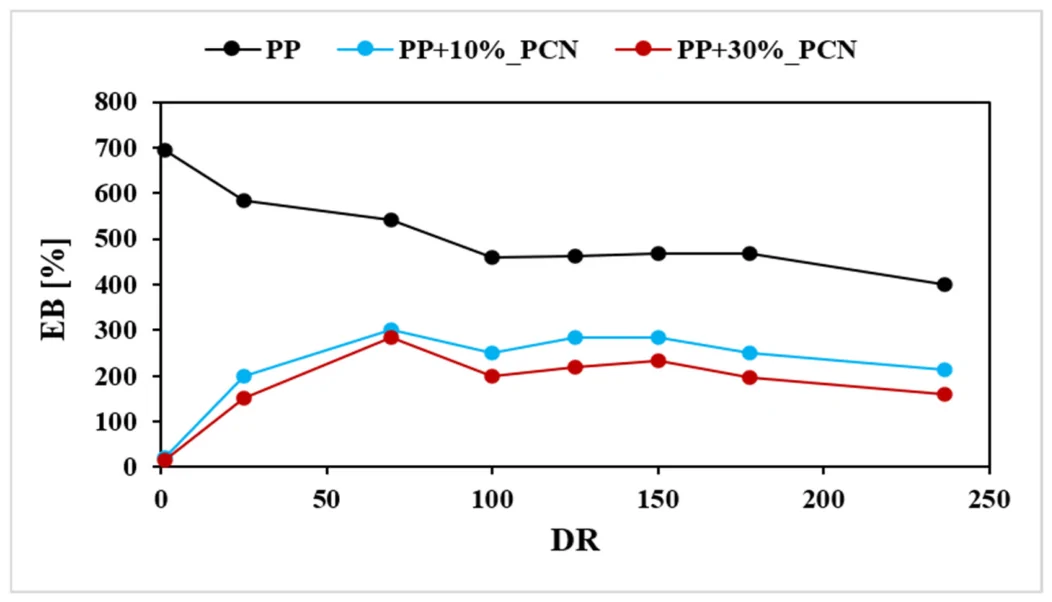
Elongation at break as a function of the draw ratio.

In Figure 11, Figure 12 and Figure 13, the values of elastic modulus, tensile strength and elongation at break are reported as a function of the draw ratio for the fibers of the blends.

As expected, both elastic modulus and tensile strength increase with the draw ratio, and the values of the two blends are slightly below those of the virgin polymer. However, the values of both characteristics are similar to those measured on a single fiber of the net. Indeed, the elastic modulus is about 2000–2200 MPa and the tensile strength is 250–300 MPa.

On the contrary, the elongation at break shows two distinct behaviors: the values relative to the ductile virgin sample decrease with increasing draw ratio; and the EB values of the two brittle blends first increase with increasing draw ratio and then decrease.

The increase in elastic modulus and tensile strength with the draw ratio is certainly due to the orientation of the macromolecules along the drawing direction. The same interpretation holds for the decrease in the elongation at break with the draw ratio of the virgin polymer. The oriented macromolecules are, of course, less deformable.

More difficult to interpret is the EB behavior shown by the blends. However, in a previous paper we have reported the same behavior for other blends and other brittle samples [29].

The orientation of the macromolecules induced by the spinning process initially improves the deformability of the blends by promoting the sliding of the polymer chains. However, beyond a certain drawing ratio (DR ~ 70), the excessive orientation of the matrix increases the brittleness of the material, leading to a reduction in the elongation at break.

## 4. Conclusions

Discarded fishing nets from marine activities were successfully mechanically recycled and reprocessed through blending with the same virgin polymer, demonstrating the potential of this approach for the valorization of post-consumer fishing nets. The incorporation of virgin PP proved to be an effective strategy to compensate for the deterioration of the material properties associated with the prolonged service life and environmental exposure experienced by the fishing nets.

Indeed, the recycled nets exhibited evident signs of degradation caused by photo-oxidative and environmental aging phenomena occurring during their use in marine environments. Furthermore, the presence of biological incrustations and other contaminants contributed to the overall deterioration of the material, negatively affecting its processability and mechanical performance. As a consequence, the post-consumer nets alone showed insufficient rheological and mechanical properties for stable fiber production.

However, when blended with virgin PP, the recycled material displayed a significantly improved processing behavior. Although it was not possible to obtain stable fibers from the recycled PCN alone, the blends with virgin PP enabled the successful production of fibers characterized by melt strength values slightly higher than those of virgin PP and breaking stretching ratio (BSR) values only slightly lower than those of virgin PP. Nevertheless, these values still remained within the acceptable range for industrial operability and fiber-processing applications.

Comparing the two formulations, PP + 30%_PCN exhibited slightly higher stiffness and strength than PP + 10%_PCN, while incorporating a larger fraction of recycled material without impairing processability, making it preferable when maximizing waste valorization is the priority; PP + 10%_PCN may instead be a more conservative choice where higher ductility is required. The present study evaluated only a single reprocessing step, and repeated reprocessing could reasonably be expected to further affect mechanical properties, although this remains to be verified experimentally.

Overall, the results demonstrated that the addition of virgin polypropylene can effectively mitigate the negative effects of degradation in post-consumer fishing nets, allowing their successful reintroduction into processing cycles, although ultimate disposal will still be necessary once material properties fall below the threshold required for further reprocessing. This strategy represents a promising route toward the development of sustainable recycling practices and circular economy approaches for marine plastic waste management.

## Figures and Tables

**Table 1:** Main characteristics of synthetic and biodegradable polymers used in fishing nets.

Property	Synthetic Polymers (PA6, PA66, HDPE, PP, PES) [5]	Biodegradable Polymers (PLA, PBS, PBAT, PHA) [6,7,8]
**Origin**	Derived from fossil resources	Mostly derived from renewable biological resources (e.g., PLA, PHA); PBAT is typically fossil-derived despite being biodegradable; PBS can be produced from either fossil or renewable feedstocks
**Biodegradability**	Generally non-biodegradable and persistent in the marine environment	Biodegradable, although the rate strongly depends on polymer type and environmental conditions (e.g., soil vs. marine)
**Mechanical performance**	High tensile strength, toughness, and abrasion resistance, ensuring reliable performance under severe marine conditions	Strongly dependent on polymer type and formulation; generally lower load-bearing capacity than conventional synthetic fibers unless enhanced through engineering approaches (e.g., nanocomposites, bioinspired architectures)
**Resistance in marine environments**	High resistance to water, salinity and prolonged exposure, contributing to long-term durability	Variable stability; several bio-based polymers show reduced long-term durability under real marine operating conditions
**Current use in fishing gear**	Dominant material choice due to proven long-term reliability, but a major contributor to marine plastic persistence	Industrial uptake in fisheries remains limited due to insufficient long-term durability and reliability under real operational conditions [9]

**Table 2:** Investigated samples with their code.

Sample	Sample Code
Post-consumer fishing net 1	NET_1
Post-consumer fishing net 2	NET_2
Post-consumer fishing net 1+ post-consumer fishing net 2; (50:50)	PCN
Virgin Polypropylene Moplen^®^ RP340H	Virgin PP
Polypropylene + 10%wt post-consumer fishing nets	PP + 10%_PCN
Polypropylene + 30%wt post-consumer fishing nets	PP + 30%_PCN

**Table 3:** Mean values of elastic modulus, tensile strength and elongation at break of NET_1 and NET_2 fibers, with corresponding standard deviations.

Samples	E [MPa]	TS [MPa]	EB [%]
NET_1	2594 ± 142	299 ± 45	75 ± 19
NET_2	2233 ± 198	264 ± 40	118 ± 15

**Table 4:** Melting temperature, melting enthalpy and crystallinity degree of the two post-consumer fishing nets.

Samples	Tm [°C]	ΔHm [J/g]	ꭓc [%]
NET_1	133.2	154.25	75
NET_2	133.5	115.68	56

**Table 5:** Mean values and corresponding standard deviations of the elastic modulus, tensile strength, and elongation at break of the isotropic samples.

Sample	E [MPa]	TS [MPa]	EB [%]
PP	438 ± 54	16 ± 1	695 ± 33
PP + 10%_PCN	483 ± 11	13 ± 5	22 ± 6
PP + 30%_PCN	495 ± 23	18 ± 5	12 ± 5
PCN	533 ± 46	18 ± 2	7 ± 1

## Data Availability

The data presented in this study are available upon request from the corresponding author.
